# Issues with the targeted therapy of non‑small cell lung cancer with thyroid metastases: A case report

**DOI:** 10.3892/mi.2023.117

**Published:** 2023-10-17

**Authors:** Ertugrul Bayram, Tugba Toyran, Burak Güney, Aysun Hatice Uguz, Derya Gümürdülü, Semra Paydas

**Affiliations:** 1 Department of Medical Oncology, Cukurova University Faculty of Medicine, Sarıçam, Adana 01330, Turkey; 2 Department of Pathology, Cukurova University Faculty of Medicine, Sarıçam, Adana 01330, Turkey; 3 Department of Nuclear Medicine, Cukurova University Faculty of Medicine, Sarıçam, Adana 01330, Turkey

**Keywords:** tumor heterogeneity, alectinib, lung cancer, treatment

## Abstract

Lung cancer is a common malignancy that has usually already metastasized at the time of diagnosis; however, thyroid metastases are extremely rare. Echinoderm microtubule-associated protein-like 4-anaplastic lymphoma kinase (ALK) fusion has been observed in 3-7% of cases of lung adenocarcinoma. ALK inhibitor therapy has been shown to exert a positive effect on disease progression. The present study describes the case of a patient with ALK-positive non-small cell lung carcinoma and thyroid metastases who exhibited a minimal response to ALK inhibitor therapy in the primary lesion, but had a complete pathological response in the thyroid, as confirmed by a thyroid biopsy. The present case report undermines the need for further evidence from genomic testing following this different tumor course in thyroid tissue.

## Introduction

Lung carcinoma has a poor prognosis, is metastatic and progresses in an aggressive manner (1,2). The majority of cases of lung carcinoma are non-small cell lung cancer (NSCLC), which are caused by a wide range of molecular alterations. In lung adenocarcinomas, an echinoderm microtubule-associated protein-like 4 (EML4)-anaplastic lymphoma kinase (ALK) fusion has been detected in 3-7% of cases. Tyrosine kinase inhibitors (TKIs) have a considerable effect on survival compared to other treatments in adenocarcinomas positive for EML4-ALK (3). Lung adenocarcinomas can be treated by targeting ALK. ALK, a transmembrane tyrosine kinase receptor from the insulin receptor superfamily that is located on the second chromosome, has been the subject of extensive studies. Following the discovery of ALK rearrangement, increasingly potent first-, second- and third-generation ALK TKIs are being developed for treatment. Patients with metastatic lung adenocarcinomas that are ALK-positive have a better prognosis and respond well to ALK inhibitor therapies. ALK inhibitors have been used in tumor-targeted medicines to achieve progression-free survival and an improved quality of life (1,4,5). Since the introduction of the first-generation ALK TKI, crizotinib, more selective second-generation (brigatinib and alectinib) and third-generation (lorlatinib) TKIs have been developed; however, resistance mechanisms complicate treatment. However, there are still numerous unresolved questions and additional research is warranted (1,4-6).

A second-generation ALK inhibitor known as alectinib is used in the treatment of metastasized or relapsed NSCLC (7,8). Thyroid metastasis is a very rare occurrence, although its incidence increases with autopsy (9). According to autopsy results, breast cancer, renal cell carcinoma and lung cancer may be the origins of thyroid metastases. Thyroid metastases have a negative impact on patient prognosis (10). Patients with lung cancer metastases to the thyroid were previously treated with chemotherapy using the drugs, cisplatin and etoposide, or with chemotherapy and radiotherapy (8).

The present study describes a rare case of lung cancer in which the metastasis in the thyroid exhibited a complete response to targeted therapy, while the main lesion responded poorly.

## Case report

**Figure 1:**
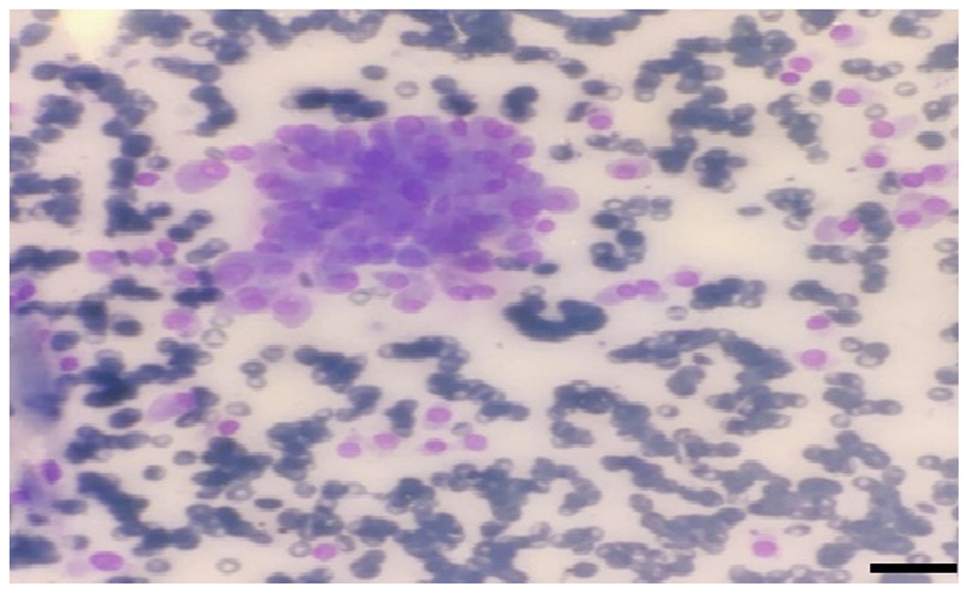
Pathological findings prior to alectinib treatment. Thyroid fine-needle aspiration biopsy revealed malignant epithelial cells with three-dimensional gland formation, extensive cytoplasm, rounded large nuclei and prominent nucleoli (Giemsa staining; x400 magnification; scale bar, 25 µm).

A 46-year-old male patient was admitted to the chest diseases clinic of Çukurova University Balcalı Hospital with dyspnea and a mass was detected in the left lower lung lobe during imaging. The patient had mediastinum, thyroid and lung involvement according to the results of whole-body positron emission tomography/computed tomography (PET-CT) (as shown below). A thyroid fine needle aspiration biopsy was performed for metastasis (Fig. 1). Giemsa staining was performed in the pathology laboratory. The May-Grünwald solution used was from MilliporeSigma. Thin needle smears for Giemsa staining were air-dried at 23-25˚C. Approximately 60 cc of May-Grünwald solution were added to a Coplin jar. The air-dried smears were immersed in the Coplin jar and left for 10 min. Another Coplin jar was prepared with ~60 cc of 1% Giemsa solution (1 cc Giemsa in 9 cc distilled water, for example). The smears were then incubated in this solution for 10 min. Subsequently, they were rinsed with tap water. Stained slides were dried using a heat source emitting mild heat at moderate temperature. Prepared slides were examined under an Olympus Bx51 light microscope (Olympus Corporation). The scan results included a spiculated contoured hypermetabolic soft tissue mass in the central part of the lower lobe of the left lung consistent with atelectasis, pleural effusion, intense focal increased metabolic activity in the left thyroid lobe and hypermetabolic lesions in other regions. Adenocarcinoma was diagnosed following a biopsy of the lung mass (data not shown).

The biopsy from the left thyroid lobe was compatible with adenocarcinoma metastasis. Video-assisted thoracoscopic surgery, left thoracotomy, lymph node biopsies and pleural biopsy were performed for the mass in the left lung, with the diagnosis of lung adenocarcinoma. EML4/ALK FISH analysis was performed. Four-micron sections were obtained from paraffin blocks of the case and mounted on positively charged slides. The sections were incubated in an oven at 56˚C overnight. They were then soaked in three separate containers, each containing xylene, for 10 min. Subsequently, they were dehydrated by being immersed twice in absolute ethanol for 5 min each time and air-dried. A water bath was set to 80˚C, and a deparaffinization pre-wash solution was placed in a heat-resistant covered container. For every five slides, a separate slide box was prepared, and it contained 15 ml of distilled water and 150 µl of 1 M HCl. These slide boxes were placed inside a water bath set at 37˚C. Following removal from the water bath, the slides were briefly dipped (10-15 sec) in distilled water at room temperature. Following this, they were immersed twice for 3 min each in 2X SSC solution. The sections were subsequently passed through a series of alcohol solutions (70, 85 and 100%) for 3 min each and then allowed to air-dry.

**Figure 2:**
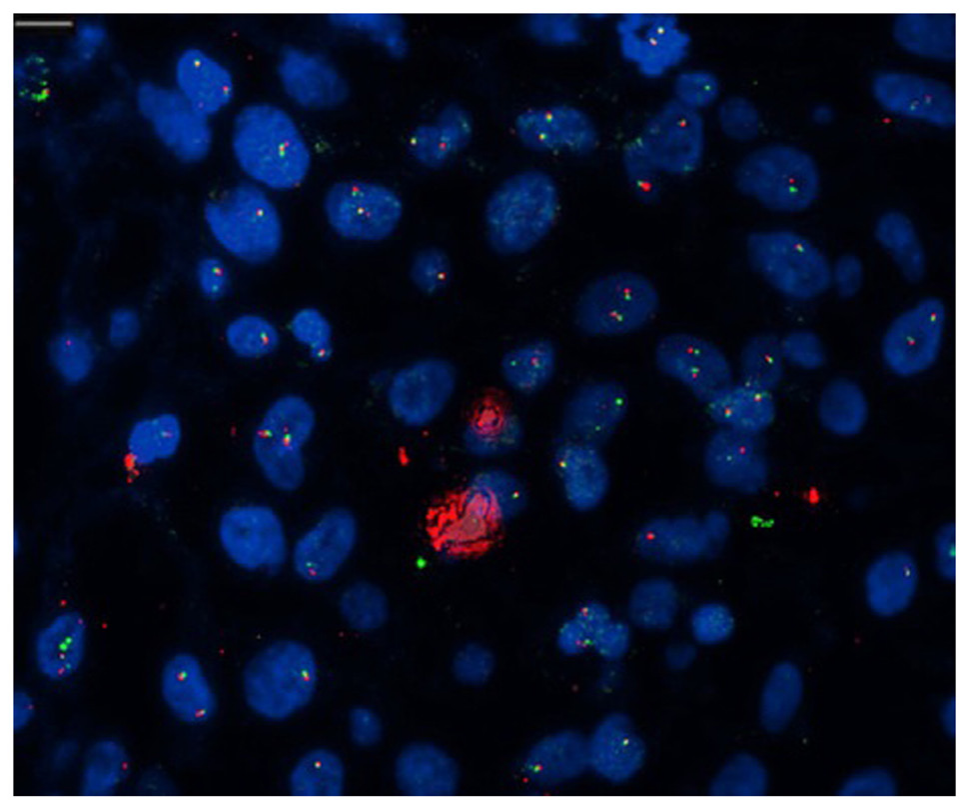
Results of FISH analysis of the echinoderm microtubule-associated protein-like 4-anaplastic lymphoma kinase gene region (scale bar, 10 µm).

After drying, 10 µl of ALK Break Apart Probe (Abbott Pharmaceutical Co. Ltd./Vysis ALK Break Apart FISH Probe kit) was applied to the sections. The slides were then cover-slipped, and the edges were sealed with rubber. Denaturation was carried out in a ThermoBrite device at 73˚C for 5 min. Hybridization was performed for 16 h at 37˚C. The following morning, the sections were incubated in a washing solution at 73˚C for 3 min, followed by a 2-min incubation at room temperature. After drying, 10 µl of DAPI was applied to the sections for counterstaining, and cover slips were placed. Prior to evaluation, the slides were stored at -20˚C for 1 h. The FISH analysis was conducted using a computer-linked fluorescent microscope (Olympus BX61; Olympus Corporation). At least 100 nuclei were counted in each slide, and separation signals (break apart) were considered present when the distance between red and green signals was at least twice the estimated signal diameter or when a single red signal was detected. The presence of separation signals in more than 15% of tumor cells was considered positive for the 2p23 translocation. Herein, as 25 out of 100 counted cells exhibited translocation, it was considered positive (Fig. 2).

**Figure 3:**
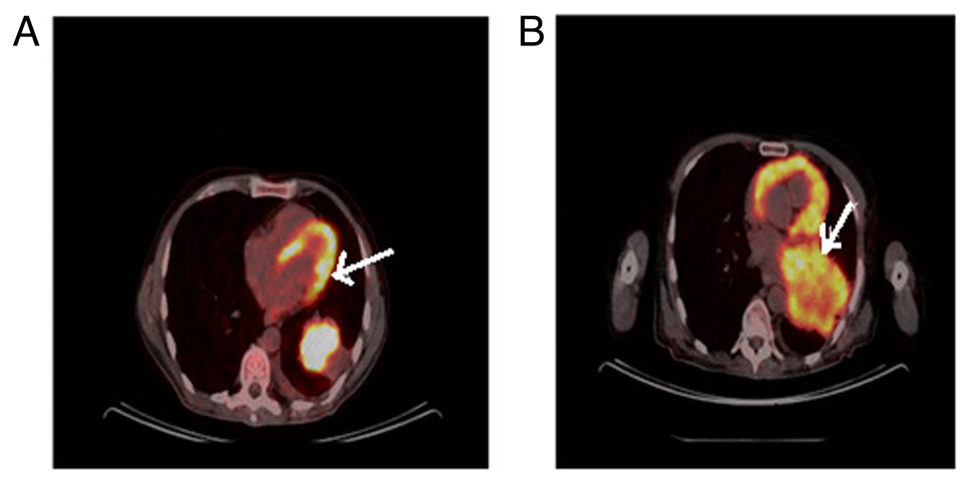
The arrows in the images indicate the progression of the primary lesion in the patient's lung. (A) Prior to alectinib treatment. (B) The primary lesion in the patient's lung had increased in size by 50% following alectinib treatment.

**Figure 4:**
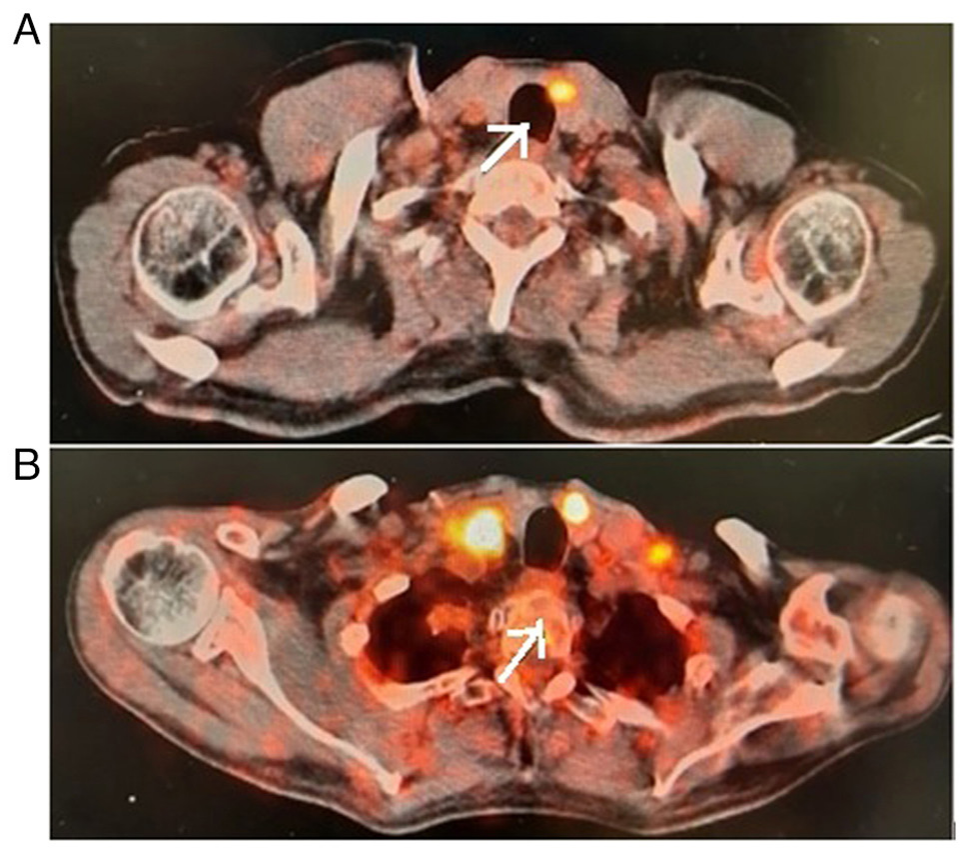
The arrows in the images indicate the radiological response following alectinib therapy. (A) Following alectinib treatment, the involvement of the thyroid and surrounding lymph nodes regressed. (B) The thyroid and surrounding lymph nodes were involved prior to alectinib treatment.

**Figure 5:**
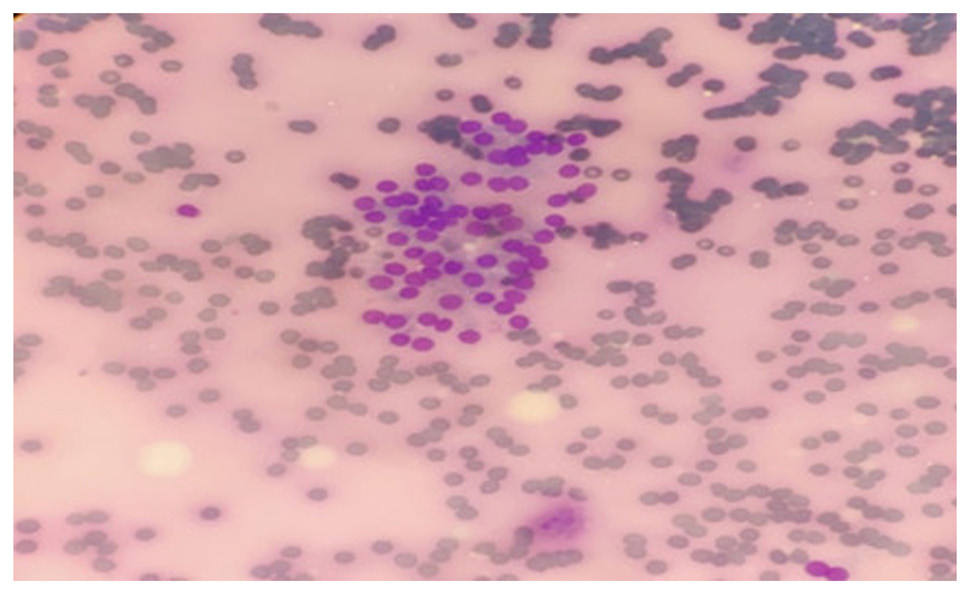
Pathological findings following alectinib treatment; thyroid follicle epithelial cells of benign appearance, arranged in microfollicles and macrofollicles (Giemsa staining; x400 magnification; scale bar, 25 µm).

The patient commenced treatment with the ALK inhibitor, alectinib. The clinical course of the patient was quite complex. Despite the treatments, the primary lesion in the lungs progressed rapidly (Fig. 3), while PET-CT imaging revealed the regression of the thyroid gland (Fig. 4). A re-biopsy from the same site showed a pathologic complete response in the thyroid (Fig. 5). In the clinical follow-up of the patient, the lesion in the lung progressed and chemotherapy (platinum and taxane) was commenced due to a visceral crisis. The patient's final condition was characterized by respiratory distress, signs of infection, and worsening blood counts. Finally, the patient's respiratory distress became severe and he was admitted to the intensive care unit of Cukurova University Hospital, where he was intubated, and eventually, succumbed.

## Discussion

The incidence of lung cancer has steadily risen due to the increased life expectancy, smoking status and environmental factors. NSCLC represents ~85% of lung cancer cases. Lung cancer is a malignancy that, at the time of diagnosis, has generally already metastasized. NSCLC metastasis to the thyroid is a very rare occurrence. Despite the extensive circulatory network of the thyroid gland, carcinomas do not often spread to the thyroid gland. Not only ALK-positive NSCLC, but also renal cell carcinomas (~50%) are the most common primary carcinomas metastasizing to the thyroid gland (2,11,12). The EML4-ALK fusion has been reported in 3 to 7% of NSCLC cases. Patients with metastatic NSCLC that have a driver mutation are treated with medications that target specific molecular pathways (13,14). ALK inhibitors have been used in tumor-targeted therapies to achieve progression-free survival and an improved quality of life. In the case described herein, tumor heterogeneity was assumed to be responsible for the poor response of the main lesion and the complete response of the metastasis in the thyroid. The small number of ALK-positive clones in the initial lesion and the ALK-positive clone that metastasizes to the thyroid may be related to this disorder. Primary thyroid tumors can also reveal ALK-positive clones, and these clones may similarly have a stronger propensity for the thyroid. Tumor heterogeneity is a key factor in the variable outcomes of treatment (15), as in the rare case of thyroid metastasis described in the present study.

The primary objective of the treatment approach used for the patient described herein was to assess the response of EML4-ALK-positive lung cancer to the ALK inhibitor, alectinib. A marked positive response in the thyroid gland and surrounding lymph node metastases was observed, as evidenced by PET-CT imaging and confirmed by a pathological examination, which indicated a pathological complete response in the thyroid.

However, despite the promising response in the thyroid gland, the main finding of the treatment was the rapid progression of the primary lung lesion. Notably, the lung lesion continued to advance even though there were no signs of lesions in the thyroid gland. This finding suggests that while alectinib was effective in controlling metastatic disease in the thyroid, it did not have the same level of success in treating the primary lung tumor. Despite advances in target-specific therapies in cases in the literature even without thyroid metastases (16,17), the majority of patients with ALK-positive NSCLC treated with TKIs ultimately experience disease progression due to various mechanisms of drug resistance (18).

As regards the patient's demise, it is crucial to clarify that treatment with alectinib was not directly associated with patient not surviving. Following the progression of the primary lung lesion and the initiation of chemotherapy (platinum and taxane) due to a visceral crisis, the patient did not respond to chemotherapy, leading to her passing away.

In summary, the main finding of the treatment was the disparate response between the thyroid gland metastases (positive response to alectinib) and the primary lung lesion (rapid progression). Furthermore, the patient's demise was primarily attributed to the progression of the primary lung lesion and the subsequent failure of chemotherapy to control it.

The percentage of ALK-positive cells may differ in tumor tissue in the thyroid and lung. The high percentage of ALK-positive cells in the thyroid tissue may have resulted in a greater benefit from alectinib therapy. Patients with varying percentages of ALK-positive cells also have varying alectinib responses. Patients with higher ALK percentages have better responses (15). The present study aimed to draw attention to the fact that the difference in ALK percentages between patients may also differ in metastatic tissues of the same patient. In the case described herein, a low ALK percentage positivity in the lung tissue may explain the weak response in the lung (19).

Next-generation sequencing (NGS) is occasionally indicated in oncology in most recent years to identify actionable targets; however, the methodology itself still requires a well-identified and sufficient number of tumor cells to perform the sequencing and an experienced team to perform the analysis. For technical reasons, the authors of the present study could not evaluate the ALK cell percentage and further analysis from thyroid tissue was not possible. Although the sample collected in the present case was sufficient to diagnose the tumor, it was insufficient for further examination. In the case described herein, only detect cancer cells could be detected. In targeted treatments with precision medicine, both liquid-based and tissue-based biopsies may need to be performed together in NGS. According to precision medicine, liquid-based and tissue-based biopsies are complementary, not an alternative to each other, in offering the optimal treatment strategy (20).

Tumor heterogeneity is a key factor that may result in inconsistent treatment outcomes. In order to improve treatment outcomes, individualized and targeted medicines that take into consideration the particular molecular characteristics of each patient's tumor may be recommended. The development of genomic and proteomic profiling techniques holds promise for the future identification of particular biological targets and the improvement of therapeutic outcomes.

There are some limitations to the present study that should be mentioned. The main limitation of the patient's data was that the percentage of ALK expression and the presence of other driver gene mutations were not investigated. In future cases, further extensive molecular investigations need to be performed.

New information on targeted treatments is continually being obtained, which is one of the most notable achievements of precision medicine. As the data on this subject increase, further light will be shed on this entity. It is clear that more advanced diagnostic methods and new strategies are required. The case in the present study is important in terms of guiding future studies on this subject and allowing clinicians to question treatment decisions based on genetic test results.

## Data Availability

The datasets used and/or analyzed during the current study are available from the corresponding author on reasonable request.
